# Supplementary material for: Clinical Pharmacists’ Knowledge of and Attitudes toward Older Adults
Source: Pharmacy (Basel). 2021 Oct 20;9(4):172. doi: 10.3390/pharmacy9040172 (PMC8544685; doi:10.3390/pharmacy9040172)
Supplement: Supplementary file 1 [file pharmacy-09-00172-s001.zip › pharmacy-1372452-supplementary.pdf]

Survey Distributed to AHS Pharmacists

1. What is your gender?
    a) Male
    b) Female
2. How old are you?

    ______ years
3. How many years have you practiced as a licensed Canadian pharmacist ?

    _______ years
4. What best describes your current practice?
    a) No contact with geriatric patients
    b) Minimal contact with geriatric patients (less than 25% of your time is spent with geriatrics)
    c) Moderate contact with geriatric patients (26-50% of your time spent with geriatrics)
    d) Significant contact with geriatric patients (greater than 50% of you time spent with geriatrics)
5. What is the extent of education you have received in geriatrics? Check all that apply.
    - None
    - Undergraduate geriatrics course
    - On the job experience training due to a large geriatric population
    - Continuing education courses

- Part of a residency program
- Certified geriatric pharmacist
- Other_____________

Select true or false for the following questions.

6. The majority of old people (past age 65) are senile (i.e. defective memory, disoriented, or demented).
7. All five senses (sight, hearing, taste, touch, smell) tend to decline in old age.
8. Most old people have no interest in, or capacity for, sexual relations.
9. Lung capacity tends to decline in old age.
10. The majority of old people feel miserable most of the time.
11. Physical strength tends to decline in old age.
12. At least one-tenth of the aged are living in long-stay institutions (i.e. nursing homes, mental hospitals, homes for the aged, etc.).
13. Aged drivers have fewer accidents per person than drivers under age 65.
14. Most older workers cannot work as effectively as younger worker.
15. About 80% of the aged are healthy enough to carry out their normal activities.
16. Most old people are set in their ways and unable to change.
17. Old people usually take longer to learn something new.
18. It is almost impossible for most old people to learn new things.
19. The reaction time of most old people tends to be slower than reaction time of younger people.
20. In general, most old people are pretty much alike.

21. The majority of old people are seldom bored.
22. The majority of old people are socially isolated and lonely.
23. Older workers have fewer accidents than younger workers.
24. Over 15% of the Canadian population are now age 65 or older.
25. Most medical practitioners tend to give low priority to the aged.
26. The majority of older people have incomes below the poverty level (as defined by the Federal Government).
27. The majority of old people are working or would like to have some kind of work to do (including housework and volunteer work).
28. Older people tend to become more religious as they age.
29. The majority of old people are seldom irritated or angry.
30. The health and economic status of old people will be about the same or worse in the year 2037, compared to young people.

For the following select the letter on the scale following each statement that is closest to your opinion:

a) Strongly disagree
b) Disagree
c) Slightly disagree
d) Slightly agree
e) Agree
f) Strongly agree

31. It would probably be better if most old people lived in residential units with people their own age.
32. It would probably be better if old people lived in residential units with younger people.
33. There is something different about old people; it’s hard to find out what makes them tick.
34. Most old people are really no different from anybody else; they’re as easy to understand as younger people.
35. Most old people get set in their ways and are unable to change.
36. Most old people are capable of new adjustments when the situation demands it.
37. Most old people would prefer to quit work as soon as pensions or their children can support them.
38. Most old people would prefer to continue working just as long as they possibly can rather than be dependent on anybody.
39. Most old people tend to let their homes become shabby and unattractive.
40. Most old people can generally be counted on to maintain a clean, attractive home.
41. It is foolish to claim that wisdom comes with age.
42. People grow wiser with the coming of old age.
43. Old people have too much power in business and politics.
44. Old people should have power in business and politics.
45. Most old people make one feel ill at ease.
46. Most old people are very relaxing to be with.

47. Most old people bore others by their insistence on talking “about the good old days.”
48. One of the most interesting and entertaining qualities of most old people is their accounts of their past experiences.
49. Most old people spend too much time prying into the affairs of others and giving unsought advice.
50. Most old people tend to keep to themselves and give advice only when asked.
51. If old people expect to be liked, their first step is to try and get rid of their irritating faults.
52. When you think about it, old people have the same faults as anybody else.
53. In order to maintain a nice residential neighbourhood, it would be best if too many old people did not live in it.
54. You can count on finding a nice residential neighbourhood when there is a sizeable number of old people living in it.
55. There are a few exceptions, but in general most old people are pretty much alike
56. It is evident that most old people are very different from one another.
57. Most old people should be more concerned with their personal appearance; they’re too untidy.
58. Most old people seem quite clean and neat in their personal appearance.
59. Most old people are irritable, grouchy, and unpleasant.
60. Most old people are cheerful, agreeable, and good humoured.
61. Most old people are constantly complaining about the behaviour of the younger generation.

62. One seldom hears old people complaining about the behaviour of the younger generation.
63. Most old people make more excessive demands for love and reassurance than anyone else.
64. Most old people need no more love and reassurance than anyone else.
